# Interesting Paragraphs

**Published:** 1895-02

**Authors:** 


### Medical News and Miscellany.

**Dr. E. R. Walker** has removed from Cistern to Weimar.

---

**Dr. Jas. W. Cole,** late of Howe, Texas, has located at Kennedale, Texas.

---

**It is "Sir"** Jno. Eric Erichsen now. He has been made a Baronet. Railway spine still lives.

---

**A bill** is now before the legislature making castration the penalty for rape or attempted rape, in accordance with what seems to be a popular demand.

---

**D. T. Terrill Jackson,** late of Iredell, one of the first graduates of the Texas Medical College, was married on the 9th inst. to Miss Mary Davis, daughter of Mrs. R. H. Smith, of Austin. The doctor will locate in Waco, we learn.

**Dr. B. F. Church**, for several years Assistant Superintendant of the North Texas Insane Asylum, at Terrell, resigned the position and has gone to Baltimore to study at the Johns-Hopkins University and at the Presbyterian Eye and Ear Hospital.

---

**Dr. F. B. King** has removed from Lampasas to Houston. Dr. King practiced four years in Burnet and six years in Lampasas; at the latter place he was surgeon of the G. & C. R. R. and President of the Board of Examiners, 27th District and also of the Pharmacy Board.

---

**Dr. Karnes.**—The JOURNAL is pleased to learn that Dr. W. C. Karnes, of Gonzales, who has been appointed Assistant Superintendent for the S. W. Texas Insane Asylum at San Antonio, and who was recently badly wounded while turkey hunting, has about recovered and will soon be able to go on duty at his post.

---

**Quarantine Appointments.**—To date of going to press the following appointments of quarantine officers have been announced: At Brazos de Santiago, Dr. A. S. Wolff, re-appointed; Sabine Pass, Dr. A. N. Perkins, re appointed; Pass Cavallo, Dr. T. J. McFarland, vice Dr. Duncan; Aransas Pass, Dr. J. D. Westervelt, vice Dr. B. W. Bristow. (Dr. Bristow, it is understood, declined the appointment in view of the contemplated reduction in salary as recommended in the governor's message; he could not accept the appointment at a reduced salary. The State loses a valuable officer in Dr. Bristow; his services during the past four years gave entire satifaction to the authorities.) At Galveston and at Velasco no appointment has yet been made. Of the inspectors on the Rio Grande, Drs. Yandell and Turpin are re-appointed and Dr. Lott is succeeded at Eagle Pass by Dr. A. H. Evans, a worthy successor and a first-class man.

